# Binary Self-Assembly of Nanocolloidal Arrays Using Concurrent and Sequential Spin Coating Techniques

**DOI:** 10.3390/ma14020274

**Published:** 2021-01-07

**Authors:** Shih-Jyun Shen, Demei Lee, Yu-Chen Wu, Shih-Jung Liu

**Affiliations:** 1 Department of Mechanical Engineering, Chang Gung University, Taoyuan 33302, Taiwan; m8420@cgmh.org.tw (S.-J.S.); dmlee@mail.cgu.edu.tw (D.L.); wusz1687@gmail.com (Y.-C.W.); 2 Department of Anesthesiology, Chang Gung Memorial Hospital-Linkou, Taoyuan 33305, Taiwan; 3 Bone and Joint Research Center, Department of Orthopedic Surgery, Chang Gung Memorial Hospital-Linkou, Taoyuan 33305, Taiwan

**Keywords:** binary colloidal array, polystyrene nanosphere, spin coating, concurrent and sequential coating, process optimization

## Abstract

This paper reports the binary colloid assembly of nanospheres using spin coating techniques. Polystyrene spheres with sizes of 900 and 100 nm were assembled on top of silicon substrates utilizing a spin coater. Two different spin coating processes, namely concurrent and sequential coatings, were employed. For the concurrent spin coating, 900 and 100 nm colloidal nanospheres of latex were first mixed and then simultaneously spin coated onto the silicon substrate. On the other hand, the sequential coating process first created a monolayer of a 900 nm nanosphere array on the silicon substrate, followed by the spin coating of another layer of a 100 nm colloidal array on top of the 900 nm array. The influence of the processing parameters, including the type of surfactant, spin speed, and spin time, on the self-assembly of the binary colloidal array were explored. The empirical outcomes show that by employing the optimal processing conditions, binary colloidal arrays can be achieved by both the concurrent and sequential spin coating processes.

## 1. Introduction

In tissue engineering and regenerative medicine, nanotechnologies can be used to fabricate biomimetic scaffolds with an increased complexity and vascularization [1,2]. The biomimetic 3D scaffold provides an excellent micro-environment for cell growth and multi-differentiation, as well as new tissue formation. In a tissue-like nano-structure, highly interconnected pores favor cell migration and vascular ingrowth. The nanofeatured surfaces with tailored wetting properties also promote cell adhesion, proliferation, alkaline phosphatase activity, and the expression of osteogenic/cementogenic-related markers [3,4]. Furthermore, through nanopatterning techniques, it is possible to precisely position selected biomolecules on a substrate for driving cell growth and, possibly, regulating cell functions.

Various methods have been proposed to manufacture porous scaffolds for bone/tissue substitutes, including solvent casting and particle leaching [5], phase separation [6], gas foaming [7], self-assembly [8], additive manufacturing [9], electrospinning [10], etc. Among these methods, the self-assembly of two-dimensional colloidal monolayers [11] has arisen as a key tool in nanotechnology for creating templates for the etching or deposition of materials in colloidal lithography, which represents an economical and easy path for surface patterning and the manufacturing of organized arrays compared with traditional serial-direct-writing methods, such as electron beam lithography. Self-assembly represents the spontaneous process of gathered particles that creates a stable and structurally distinct aggregation by non-covalent bonds under balanced conditions. It is ubiquitous in biological systems and can be considered fundamental for creating complex biological structures. Self-assembled biomaterials mimicking the extracellular matrix (ECM) can offer a tissue-like micro-environment for cell proliferation and differentiation, thus promoting tissue regeneration [3].

Spin coating is one of the simplest methods for creating a film on a substrate [12]. It is a solution-based process that was developed for the low-cost deposition of thin films of various materials. The spin coating process begins with mixing of the material to be deposited with a solvent. The solution is then dispensed on the substrate surface and spun at a high speed. The thickness of the film can be adjusted by the spin speed, surface tension, and viscosity of the solution. The solvent is partially relieved in the spin process owing to evaporation and partially relieved by subsequent heat at raised temperatures, thus resulting in a fairly planar surface.

This study explored the self-assembly of binary colloidal arrays [13,14,15], as a template for making nanofeatured scaffolds for potential applications in tissue engineering and regenerative medicine, by means of either the concurrent or sequential spin coating technique. Polystyrene (PS) nanospheres of two dimensions, namely 900 and 100 nm, were employed. The influence of the processing parameters, including the type of surfactant solution, spin speed, and spin time, on the self-assembly of binary colloidal arrays were investigated.

## 2. Materials and Methods

### 2.1. Materials

Commercially available polystyrene colloidal nanospheres of two difference sizes, including 900 and 100 nm, were acquired from micro-particles GmbH (Berlin, Germany). Other materials, including surfactant Triton X-100, methanol, and ethanol, were purchased from Sigma-Adrich (St. Louis, MO, USA).

### 2.2. Experimental Setup

In order to obtain hydrophilic surfaces, the silicon substrates were firstly cleaned with 1:1:5 NH_4_OH/H_2_O_2_/H_2_O (*v*/*v*/*v*) at 80 °C for 15 min. Following a wash with distilled water, the substrate was handled with 1:1:6 HCl/H_2_O_2_/H_2_O (*v*/*v*/*v*) at 75 °C for 20 min, followed by piranha etched with 3:1 (*v*/*v*) H_2_SO_4_/H_2_O_2_ at 130 °C for 15 min, so as to enhance its wettability and smoothness.

**Figure 1:**
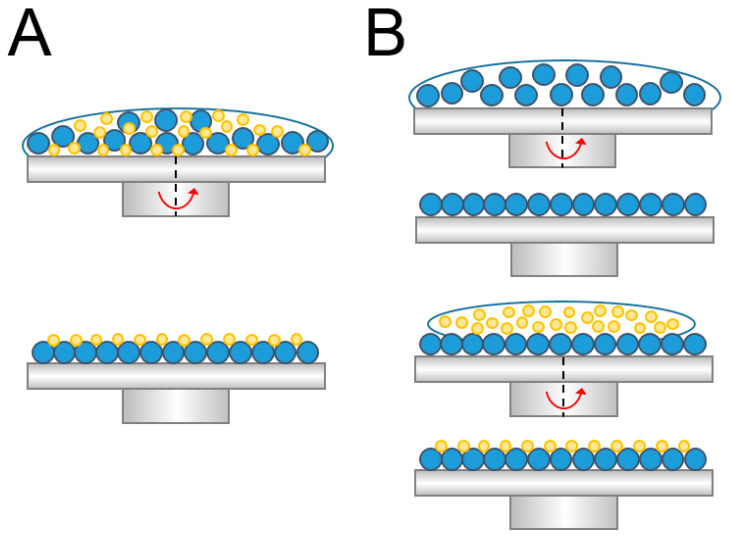
Schematic figure of (**A**) concurrent and (**B**) sequential spin coating of a binary colloidal array.

To self-assemble the binary colloid arrays, two different spin coating processes were employed, namely concurrent coating and sequential coating. Figure 1A,B schematically show the concurrent and sequential coating processes, respectively. For the concurrent spin coating, 900 and 100 nm colloidal nanosphere latexes were first mixed and spin coated on the substrate, employing a commercial spin coater (SP-M3-P, Apisc Co., Taiwan). On the other hand, the sequential coating process first created a monolayer of 900 nm nanosphere arrays on the silicon substrate, followed by the spin coating of another layer of 100 nm arrays on top of the 900 nm arrays.

### 2.3. Concurrent Spin Coating

To concurrently spin coat the binary colloid arrays, PS nanospheres of 900 and 100 nm were mixed with the surfactant at a ratio of 900 nm:100 nm:surfactant = 0.7:0.3:1 (*v*/*v*/*v*/). Various test trials were completed so as to identify the optimum processing conditions.

As shown in Table 1, the influence of the processing parameters, including the type of surfactants, spin time, and spin speed, on the spun array properties were investigated. The latex solution from the manufacturer was diluted in mixtures, namely a mixture of surfactant Triton X-100:methanol (1:300, *v*/*v*), a mixture of surfactant Triton X-100:methanol (1:400, *v*/*v*), and a mixture of distilled water:ethanol (1:1, *v*/*v*). To spin coat the arrays, three stages of spin speed (rpm)/spin (sec) were employed. The spin speeds were set at 500, 1000, 1500, 2000, and 3000 rpm, while the spin times were set at 5, 30, 60, 100, and 300 seconds.

### 2.4. Sequential Spin Coating

To spin coat the binary colloid arrays sequentially, PS nanospheres of 900 nm were mixed with the surfactant at a ratio of 0.7:0.5 (*v*/*v*), while the colloidal nanospheres of 100 nm were mixed with the surfactant at a ratio of 0.3:0.5 (*v*/*v*). Six test trials were completed to find out the conditions that led to the optimum colloidal arrays.

The coating process was completed in two stages. In stage one, the 900 nm spheres were first spin coated on the substrate, followed by stage two, which coated 100 nm spheres on top of the arrays produced in stage one. Table 2 lists the parameters used in the sequential spin coating process.

### 2.5. Characterization of Self-Assembled Binary Arrays

The ordering of the colloidal nanospheres on the silicon substrates was examined by a field-emission scanning electron microscope (Jeol Model JSM-7500F, Tokyo, Japan). Furthermore, an atomic force microscope (AFM 5100, Agilent Tech., Santa Clara, CA, USA) was employed to measure the surface structure of the binary arrays.

## 3. Results and Discussion

### 3.1. Concurrent Spin Coating of Binary Nanosphere Arrays

In this study, nanospheres of two sizes were selected, namely 900 and 100 nm. During self-assembly, attractive capillary forces, convective transport of the nanospheres, the concentration of the colloid suspension, and centrifugal force all play critical roles in determining the ordering and quality of the obtained arrays. With a too high volume ratio, nanospheres may overlap with one another during spin coating. Conversely, with a too low nanosphere volume, there might not be enough colloids to cover the entire substrate [16]. The spin coated substrate thus exhibited a sparse nanosphere distribution. Some preliminary experimental tests were first conducted to identify the appropriate amounts of spheres of each size in the solution. Excessively small spheres are apt to fully embed the larger particles in some regions, while keeping the pattern mostly intact in other regions [17]. Furthermore, an increment in the small particle cluster may result in a pattern with a substantial number of smaller particles gathered around large ones, filling all the interstitial sites/channels and fully encompassing larger particles. Therefore, the ratio of 0.7:0.3 for the 900 and 100 nm spheres, respectively, was adopted.

The influence of the processing parameters on the concurrent self-assembly of binary nanosphere arrays, including the spin time, spin speed, and type of surfactant solution, were explored. Table 1 shows the employed processing parameters and values. By altering one parameter in each experimental trial while keeping the others unchanged, we could comprehend the influence of the parameter on the self-assembly of binary nanosphere arrays.

**Figure 2:**
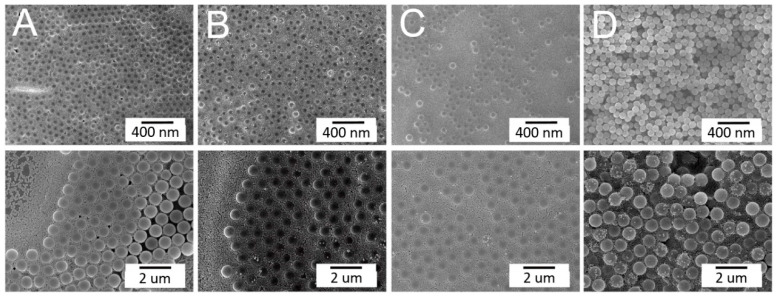
Influence of the spin time on the concurrent assembly of the binary nanosphere assay: (**A**) 500(5)/1500(30)/2000(300); (**B**) 500(30)/1500(60)/2000(60); (**C**) 500(30)/1500(60)/2000(300); and (**D**) 500(30)/1500(60)/2000(100) rpm(sec) (magnification: upper × 5000, lower × 10,000).

**Figure 3:**
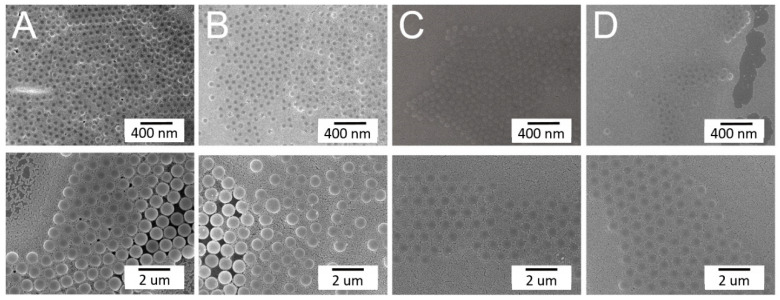
Influence of the spin speed on the concurrent assembly of the binary nanosphere assay: (**A**) 500(5)/1500(30)/2000(300); (**B**) 500(5)/1000(30)/1500(300); (**C**) 500(5)/1000(30)/2000(300); and (**D**) 500(5)/1500(30)/3000(300) rpm(sec) (magnification: upper ×5000, lower ×10,000).

Figure 2 shows the effect of the spin time on the arrays. Comparable to all naturally arising crystals, the assembled colloidal arrays exhibited diverse defects that stemmed from nanosphere polydispersity, site randomness, point and line defects, etc. [13]. Meanwhile, Figure 3 shows the characteristic of binary arrays subjected to various spin speeds. The array spin coated with 500/1500/2000 rpms in Figure 3A exhibits a more uniform nanosphere distribution characteristic. The experimental results suggest that adopting a short spin time or a slower spin speed generally leads to colloidal arrays with more uniformly distributed nanospheres on the substrates. During spin coating, the centrifugal force critically affects the quality of self-assembled arrays. The processing conditions used for the rapid deposition of larger particles may not be appropriate for ordering the smaller more movable particles that are generally deposited at considerably lower rates. An appropriate combination of the spin speed and spin time is thus important to self-assemble binary PS nanospheres with a good array quality.

**Figure 4:**
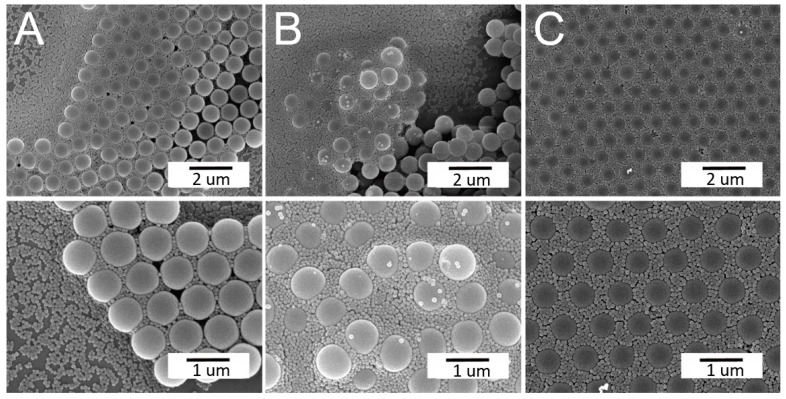
Influence of the type of surfactant solution on the self-assembly of the binary nanosphere assay: (**A**) TX-100:methanol = 1:300; (**B**) TX-100:methanol = 1:400; and (**C**) distilled water:ethanol = 1:1 (magnification: upper ×5000, lower ×10,000).

The influence of the surfactant type on spun arrays is shown in Figure 4. While the arrays spun employing TX-100 and methanol mixtures exhibited defects, such as site randomness and vacancies, the colloidal arrays prepared using the distilled water/ethanol solution displayed more uniform binary nanosphere ordering. This might be due to the fact that, relative to the TX-100/methanol mixture, distilled water/ethanol has a lower viscosity that facilitates convective transport of the nanospheres. It is thus easier for the nanospheres to be assembled in an ordered manner during the spin coating process.

### 3.2. Sequential Spin Coating of Binary Nanosphere Arrays

It has been proposed that the constitution of the binary pattern is defined by the selection of a small to large particle size ratio and their relative quantities in the mixture. Wang and Mohwald [18] proposed a stepwise spin coating method, where the layers of large (442 nm) and small (222 nm) nanospheres were created consecutively by spin coating. In this study, to self-assemble the binary nanosphere arrays, a two-stage spin coating was adopted. In the first stage, PS nanospheres of 900 nm were first spin coated on the surface of a silicon substrate. This was followed by the spin coating of 100 nm nanospheres in the second stage. The surfactant solution of the distilled water/ethanol mixture (1:1 *v*/*v*) was employed, based on the results in Section 3.1. Preliminary experimental trials were firstly conducted to identify the appropriate amounts of spheres of each size in the solutions. A ratio of 0.7:1 for the 900 nm:surfactant solution in the first stage and a ratio of 0.3:1 for the 100 nm:surfactant solution in the second stage were adopted.

**Figure 5:**
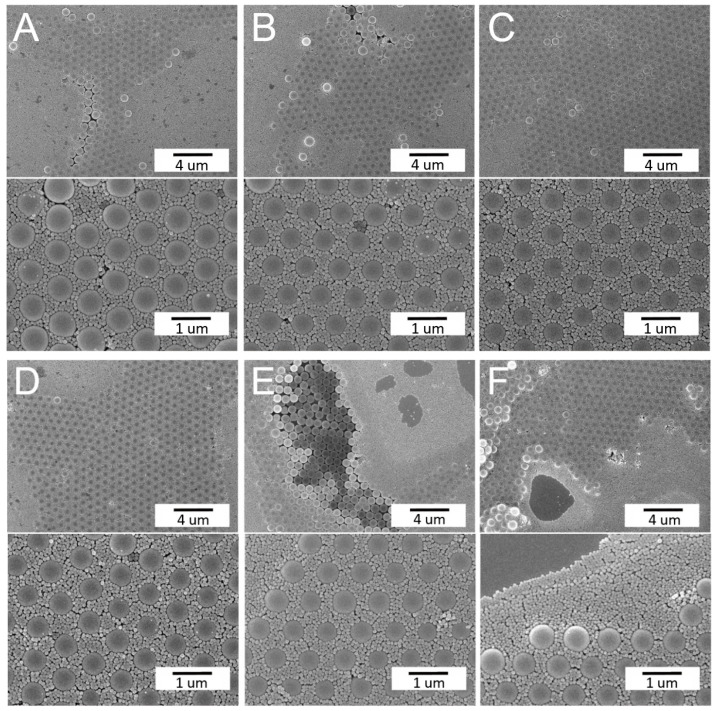
Sequentially self-assembled binary nanosphere arrays using different spin speeds/times (images **A–F** correspond to test 1–6 in Table 2) (magnification: upper × 5000, lower ×10,000).

Based on the results obtained in Section 3.1, an appropriate combination of the spin speed/spin time is key for a successful binary array assemble. The influence of spin speed/spin time consolidation was examined, and Table 2 lists the values used in the test trials. Figure 5 shows the images of self-assembled binary colloid arrays subjected to these processing conditions. Clearly, the adoption of the speeds of Test 3 in Table 2, namely 500(30)/1500(30)/2000(60) rpm(sec) in the first stage and 500(30)/3000(60)/1000(60) rpm(sec) in the second stage, assembled the binary colloidal arrays with the most uniform distribution. During the self-assembly of colloidal nanospheres, the attractive capillary forces, convective transport of the nanospheres, concentration of the colloid suspension, and centrifugal force all play a central role in defining the ordering and quality of the obtained arrays. Relative to those consisting of single-size colloidal particles, binary colloidal crystals display a rather rich phase behavior that relies on the percentages of the small and large colloidal particles, as well as on the size ratios of the small to large particles. Mukhopadhyay et al. [19] reported a self-assembly method for generating hexagonally ordered colloidal crystal nanopatterns on hydrophobic surfaces from low volume fraction dispersions. Singh et al. [20] investigated the highly ordered nanometer-scale chemical and protein patterns by binary colloidal crystal lithography combined with plasma polymerization. A rubber ring was used to confine the suspension of colloidal nanoparticles for patterning. Kitaev and Ozin [21] proposed an accelerated evaporation-induced co-assembly of binary dispersions, and pointed out that the architectures of binary colloidal arrays can be determined by the size and concentration ratios of the microspheres in the dispersions. Again, the quick deposition conditions appropriate for assembling larger particles may not be suitable for ordering the smaller particles. An appropriate combination of the spin speed and spin time is thus important for self-assembling PS nanospheres with a good array quality.

### 3.3. Self-Assembled Arrays using Optimum Parameters

**Figure 6:**
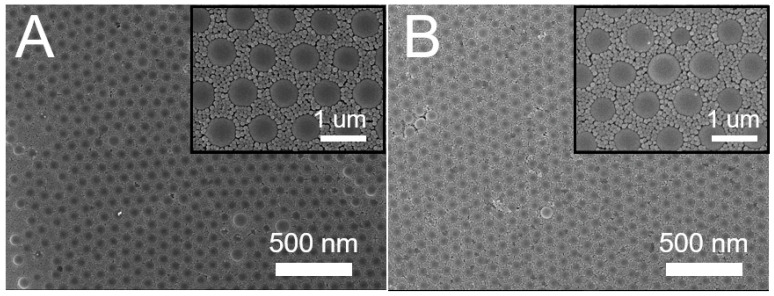
Scanning electron microscope (SEM) images of (**A**) concurrently and (**B**) sequentially assembled arrays using the optimum processing conditions.

**Figure 7:**
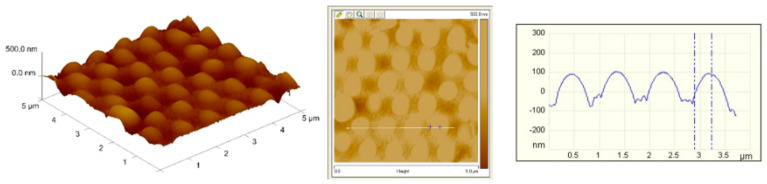
Atomic force microscope (AFM) surface profile of self-assembled binary colloid arrays.

With a combination of proper processing conditions, binary nanosphere arrays of a good dimensional uniformity, either concurrently or sequentially self-assembled, can be obtained, as shown in Figure 6. The binary arrays were characterized by an atomic force microscope (AFM), and Figure 7 shows the image of the spin coated binary arrays. The measured height ranges are 200/30 nm, which are smaller than the nominal radii of the PS nanospheres (i.e., 450/50 nm). This might be due to the fact that the 900 nm nanospheres are surrounded by the 100 nm spheres, which in turn restricts the probe tip of AFM to reach the concave area of the arrays. The measured heights decreased accordingly. Nevertheless, the empirical outcomes suggest that the spin coating technique can successfully self-assemble the nanospheres onto the silicon substrate with uniform distributions.

This study has successfully assembled binary colloidal arrays using concurrent and sequential spin coating techniques. The arrays can be employed as a template for nanostructured polymer scaffolds. In tissue engineering, cells react to nanofeatures with different chemistries and topographies, leading to variations in cell alignment, polarization, elongation, migration, proliferation, and gene expression. Through nanopatterning techniques, it is feasible to precisely locate selected biomolecules on a substrate for driving cell growth and, additionally, regulating cell functions. Other applications of these nanostructures include imaging (biolabeling and biosensing) and drug or gene delivery (e.g., nanobodies or affibodies against cancer cells) [4]. A nanofeatured surface can thus enhance the overall performances of biomaterials, promoting their use in clinical applications.

Finally, it should be noted that despite this study having successfully self-assembled the binary nanosphere arrays, the area of assembled arrays was relatively small. The self-assembly of large-area nanofeatured arrays [22] via spin coating remains a challenge, and will be the topic of our future studies.

## 4. Conclusions

This paper has reported a simple yet effective scheme for the self-assembly of binary nanosphere arrays, using concurrent and sequential spin coating. Polystyrene nanospheres of two different nanosizes (900 and 100 nm) were assembled on silicon substrates using a spin coater, either concurrently or sequentially. The assembled arrays were characterized using a scanning electron microscope (SEM) and atomic force microscope (AFM). The experimental results demonstrate that both the concurrent and sequential spin coating techniques can successfully self-assemble the nanospheres on the silicon substrate with uniform distributions. The proposed spin coating methods show great potential for the effective self-assembly of binary nanosphere arrays, due to their simplicity and versatility.

## Figures and Tables

**Table 1:** Processing parameters utilized for the concurrent spin coating.

Test No.	Surfactant:Solvent	Spin Speed (Spin Time) Unit: rpm (s)
1	TX-100:Methanol = 1:300	500(5)/1500(30)/2000(300)
2		500(30)/1500(60)/2000(60)
3		500(30)/1500(60)/2000(300)
4		500(30)/1500(60)/2000(100)
5	TX-100:Methanol = 1:300	500(5)/1500(30)/2000(300)
6		500(5)/1000(30)/1500(300)
7		500(5)/1000(30)/2000(300)
8		500(5)/1500(30)/3000(300)
9	TX-100:Methanol = 1:300	500(5)/1500(30)/2000(300)
10	TX-100:Methanol = 1:400	
11	DI water:Ethanol = 1:1	

**Table 2:** Processing parameters employed for the sequential spin coating.

Test No.	First Coating: 900 nm 900 nm: Surfactant = 0.7:1	Second Coating: 100 nm 100 nm: Surfactant = 0.3:1
	Spin Speed (Spin Time) rpm (s)	Spin Speed (Spin Time) rpm (s)
1	500(30)/1500(30)/2000(60)	500(5)/1500(30)/2000(300)
2	500(30)/1500(30)/2000(60)	500(30)/1500(30)/2000(60)
3	500(30)/1500(30)/2000(60)	500(30)/3000(60)/1000(60)
4	500(5)/1500(30)/2000(300)	500(5)/1500(30)/2000(300)
5	500(5)/1500(30)/2000(300)	500(30)/3000(60)/1000(60)
6	500(30)/3000(60)/1000(60)	500(30)/3000(60)/1000(60)

The surfactant used was distilled water:ethanol = 1:1.

## Data Availability

The data presented in this study are available on request from the corresponding author.
